# Enhancing verbal episodic memory in older and young subjects after non-invasive brain stimulation

**DOI:** 10.3389/fnagi.2013.00049

**Published:** 2013-09-11

**Authors:** Rosa Manenti, Michela Brambilla, Michela Petesi, Clarissa Ferrari, Maria Cotelli

**Affiliations:** ^1^Istituto di Ricovero e Cura a Carattere Scientifico Centro San Giovanni di Dio FatebenefratelliBrescia, Italy; ^2^Center for Cognitive Science, Department of Psychology, University of TurinTurin, Italy

**Keywords:** tDCS, aging, verbal retrieval, dorsolateral prefrontal cortex, parietal cortex

## Abstract

Memory is the capacity to store, maintain, and retrieve events or information from the mind. Difficulties in verbal episodic memory commonly occur in healthy aging. In this paper, we assess the hypothesis that anodal transcranial direct current stimulation (tDCS) applied over the dorsolateral prefrontal cortex (DLPFC) or over the parietal cortex (PARC) could facilitate verbal episodic memory in a group of 32 healthy older adults and in a group of 32 young subjects relative to a sham stimulation using a single-blind randomized controlled design. Each participant underwent two sessions of anodal tDCS (left and right) and one session of sham stimulation. Overall, our results demonstrated that, in young and in older subjects, anodal tDCS applied during the retrieval phase facilitates verbal episodic memory. In particular, we found that tDCS applied over the left and right regions (DLPFC and PARC) induced better performance in young participants; only tDCS applied over the left regions (DLPFC and PARC) increased retrieval in older subjects. These results suggest that anodal tDCS can be a relevant tool to modulate the long-term episodic memory capacities of young and older subjects.

## INTRODUCTION

Episodic memory is a fundamental form of long-term memory that relies on different processes to encode, consolidate, and retrieve information (Tulving, 1983). Several studies have shown that aging is associated with decline in the encoding and retrieval of episodic information from previously experienced events (Tulving, 1983; Spencer and Raz, 1995; Balota et al., 2000). These reductions in memory performance most likely reflect age-related changes in the brain, which undergoes significant structural and functional modifications during the aging process (Creasey and Rapoport, 1985). These age-related modifications, which are characterized by reduced activity in the networks dedicated to performing a given function, may be due to decreased cell metabolism (Burke and Barnes, 2006). Based on these age-related changes, an amendment to the hemispherical encoding retrieval asymmetry (HERA) theory (Tulving et al., 1994) was proposed for older adults. The HERA model predicts that in younger adults, the left prefrontal cortex (PFC) would specialize in encoding, while the right PFC would be crucial for retrieval. In older adults, the hemispheric asymmetry reduction (HAROLD) model has been proposed based on functional magnetic resonance imaging (fMRI) studies (Cabeza, 2002). Although the activation of the right PFC during retrieval is less pronounced, bilateral involvement of the PFC during both encoding and retrieval has been repeatedly observed in healthy older adults. Furthermore, dorsolateral prefrontal cortex (DLPFC) lateralization seems to be influenced not only by the process (encoding/retrieval) but also by the material used (verbal/non-verbal) and by the task demand (Kapur et al., 1996; Fletcher et al., 1998; Wagner et al., 1998; Poldrack et al., 1999; Golby et al., 2001).

There are numerous studies establishing the crucial role of the DLPFC in episodic memory; neuroimaging studies have demonstrated the involvement of a distributed neural network formed by the DLPFCs, the medial temporal lobes, the parietal cortices (PARCs) and the precuneus (Rugg and Wilding, 2000; Buckner et al., 2001; Fletcher and Henson, 2001; Cabeza and Nyberg, 2003; Cabeza et al., 2003, 2008; Simons and Spiers, 2003; Berryhill, 2012). Interestingly, processing of abstract and concrete words has been reported to involve different prefrontal and parietal areas (Binder et al., 2005; Klostermann et al., 2008).

Transcranial direct current stimulation (tDCS) is a non-invasive brain stimulation technique that induces long-lasting, stimulation-polarity-dependent excitability shifts in the cerebral cortex (Nitsche and Paulus, 2000, 2001; Nitsche et al., 2003a, b, 2008; Dayan et al., 2013). Recently, tDCS has facilitated memory capacity in young subjects (Boggio et al., 2009a; Chi et al., 2010; Penolazzi et al., 2010; Javadi and Cheng, 2012; Javadi and Walsh, 2012; Javadi et al., 2012; Jacobson et al., 2013) and in patients with Alzheimer’s disease (Ferrucci et al., 2008; Boggio et al., 2009b, 2011, 2012). In older subjects, some studies reported improvements in learning (Floel et al., 2012; Zimerman et al., 2013) and working memory (Berryhill and Jones, 2012) after non-invasive brain stimulation. However, no studies have explored the effects of aging on verbal episodic memory using tDCS.

The aim of this study is to assess whether anodal tDCS results in an improvement of episodic memory performance in older and young subjects. Therefore, we compared the retrieval of abstract and concrete words in both young and older subjects during the application of either anodal or placebo tDCS over the DLPFCs and PARCs. The choice of DLPFCs and PARCs as the main sites of stimulation was based on results of previous studies which showed the involvement of these two areas in episodic memory tasks (Manenti et al., 2010; Berryhill, 2012). Additionally, this work aimed to gather more information about the role of the PARC and DLPFC in episodic memory. Finally, the comparison of retrieval performances in older and young subjects, allows us to investigate the potential functional compensation of age-related changes in hemispherical asymmetry.

## MATERIALS AND METHODS

### PARTICIPANTS

Thirty-two healthy young volunteers [mean age = 23.72 ± 3.15 years; mean education = 15.13 ± 2.04 years (9 males, 23 females)] and 32 healthy older individuals [mean age = 67.91 ± 4.72 years; mean education = 10.75 ± 4.63 years (15 males, 17 females)] took part in the experiment. All of the subjects had normal or corrected-to-normal vision and were native Italian speakers. See **Table 1** for demographic details.

**Table 1:** Demographic characteristics of young and older individuals grouped according to area of stimulation.

	Young subjects (*n* = 32)			Older subjects (*n* = 32)		
	DLPFC	PARC	*p*-Value	DLPFC	PARC	*p*-Value
**Age (years)**	23.5 ± 2.2	23.9 ± 4.0	ns	67.6 ± 4.7	68.2 ± 4.9	ns
**Education (years)**	15.1 ± 2.0	15.2 ± 2.1	ns	10.0 ± 4.8	11.5 ± 4.5	ns
**EHI (%)**	57.6 ± 61.1	58.2 ± 61.4	ns^#^	88.3 ± 12.8	85.4 ± 13.3	ns

*DLPFC, dorsolateral prefrontal cortex stimulation; PARC, parietal cortex stimulation; EHI, Edinburgh Handedness Inventory; *p*-value of parametric (*t*-test) or non-parametric (Mann–Whitney test); ns, not significant.*

# *p*-Value of non-parametric Mann–Whitney test.

Participants reported being free of neurological disorders and had no history of seizures. All participants were informed about the procedures and the possible risks of tDCS, and written informed consent was obtained after a safety screening. The experimental methods got ethical approval from the local Human Ethics Committee (CEIOC – Ethics Committee of the IRCCS Centro San Giovanni di Dio Fatebenefratelli, Brescia, Italy). Prior to being enrolled in the experiment, older subjects completed a Mini Mental State Examination (MMSE; Folstein et al., 1975) and a detailed neuropsychological evaluation to verify the absence of any cognitive deficit. A pathological score in one or more of the tests was an exclusion criterion. The neuropsychological test battery included measures used to assess non-verbal reasoning (Raven’s Colored Progressive Matrices), verbal fluency (phonemic and semantic), visuo-spatial capacity (Rey–Osterrieth Complex Figure, Copy), upper-limb apraxia (De Renzi et al., 1980), attention and executive functions (Trail Making Test A and B). In addition, memory was assessed in depth (Story Recall, Rey–Osterrieth Complex Figure Recall, Digit Span, Auditory Verbal Learning Test learning and recall). All of the tests were administered and scored according to standard procedures (Lezak et al., 2004). The results of the cognitive assessments are presented in **Table 2**.

**Table 2:** Neuropsychological assessment of older subjects grouped according to area of stimulation.

	DLPFC older subjects (*n* = 16)	PARC older subjects (*n* = 16)	*p*-Value	Cut-off^*^
**Screening for dementia**		
Mini Mental State Examination	28.81 ± 1.22	28.44 ± 1.15	ns	>24
**Non-verbal reasoning**		
Raven-Colored Progressive Matrices	32.16 ± 3.64	32.56 ± 3.54	ns	>17.5
**Memory**		
Story Recall	12.44 ± 3.98	11.28 ± 4.63	ns	>7.5
Rey auditory-Verbal Learning Test-Immediate Recall	44.31 ± 8.44	44.13 ± 12.13	ns	>28.52
Rey Auditory-Verbal Learning Test-Delayed Recall	9.81 ± 2.83	8.69 ± 3.70	ns	>4.68
Rey–Osterrieth Complex Figure-Recall	12.88 ± 4.67	13.75 ± 6.88	ns	>9.46
Digit Span	5.56 ± 0.81	5.94 ± 0.68	ns	>3.5
**Praxis**		
Rey–Osterrieth Complex Figure-Copy	30.25 ± 3.97	32.34 ± 2.39	ns	>28.87
Ideomotor apraxia-right upper limb	69.38 ± 1.54	70.00 ± 1.63	ns	>62
Ideomotor apraxia-left upper limb	70.50 ± 1.26	70.69 ± 1.66	ns	>62
**Attentional and Executive functions**		
Trail Making Test A	45.31 ± 15.88	36.31 ± 11.77	ns	<93
Trail Making Test B	114.19 ± 29.31	111.56 ± 46.52	ns	<282
Trail Making Test B–A	68.88 ± 23.49	75.13 ± 38.31	ns^#^	<186
**Language**		
Fluency-Phonemic	39.75 ± 9.38	38.81 ± 11.14	ns	>16
Fluency-Semantic	43.38 ± 5.90	45.38 ± 11.17	ns	>24

*DLPFC, dorsolateral prefrontal cortex stimulation; PARC, parietal cortex stimulation; *p*-value of parametric (t-test) or non-parametric (Mann–Whitney test); ns, not significant.*

* Cut-off scores according to Italian normative data are reported. Raw scores are reported;

# *p*-Value of non-parametric Mann–Whitney test.

### STIMULI

The experimental procedure was structured in a first encoding phase followed by a retrieval phase. For the encoding phase we selected, 51 abstract and 51 concrete words from the “Corpus e Lessico di Frequenza dell’Italiano Scritto (CoLFIS)” (Laudanna et al., 1995; Bertinetto et al., 2005). For the retrieval phase, we selected an additional pool of 51 abstract and 51 concrete “new” words. Six words (three abstract and three concrete) were assigned to a practice list; the other words were divided into three experimental blocks. Thus, the retrieval word list consisted of the original encoding or “old” words (48 concrete, 48 abstract) and 96 new words (48 concrete, 48 abstract). On average, the words were 6.8 (±1.7) letters long with 2.9 (±0.7) syllables. Abstract and concrete words were balanced according to word length and to variables known to influence memory performance, i.e., word frequency and familiarity. There were significant differences between the two word categories with respect to “concreteness” (concrete = 6.3 ± 0.7; abstract = 3.9 ± 0.8; *p* < 0.05) and “imageability” (concrete = 5.9 ± 0.5; abstract = 3.3 ± 0.6; *p* < 0.05) based on CoLFIS.

### PROCEDURE

#### Task procedure

Subjects were seated in a dimly lit room, facing a computer monitor that was placed 60 cm from the subject. The stimuli were presented using Presentation software (Version 14.9, ) running on a personal computer with a 17-inch screen. Before starting the experiment, subjects completed a practice run that involved encoding and retrieval of three abstract and three concrete words. Both the encoding and the retrieval phases consisted of three separate blocks of 32 (16 concrete and 16 abstract) trials each. The three blocks were matched for familiarity, frequency, concreteness, imageability and word length (*p* > 0.05).

***Encoding phase***. During the encoding phase, subjects were presented with a word for 2000 ms, followed by an inter-trial interval of 3000 ms. For each trial, subjects were requested to indicate whether a concrete or an abstract word was presented (left index corresponded to concrete words) by pressing one of two buttons of a response box using both hands. During this phase, subjects were also requested to encode the presented words. The encoding phase was followed by a 5-min delay before the retrieval phase began.

***Retrieval phase***. During the retrieval phase, the words presented in the previous encoding block (“old”) and the “new” words were displayed in a randomized order. Each word remained on the screen until the subject provided a response. Subjects were instructed to indicate whether the word was “old” or “new” by pressing the right or left button of the button box as soon as possible. For half the subjects, the right button corresponded to “old” choice. Each response was followed by a 2000-ms delay.

In both the encoding and retrieval periods, accuracy and reaction times (RTs) were collected.

**FIGURE 1:**
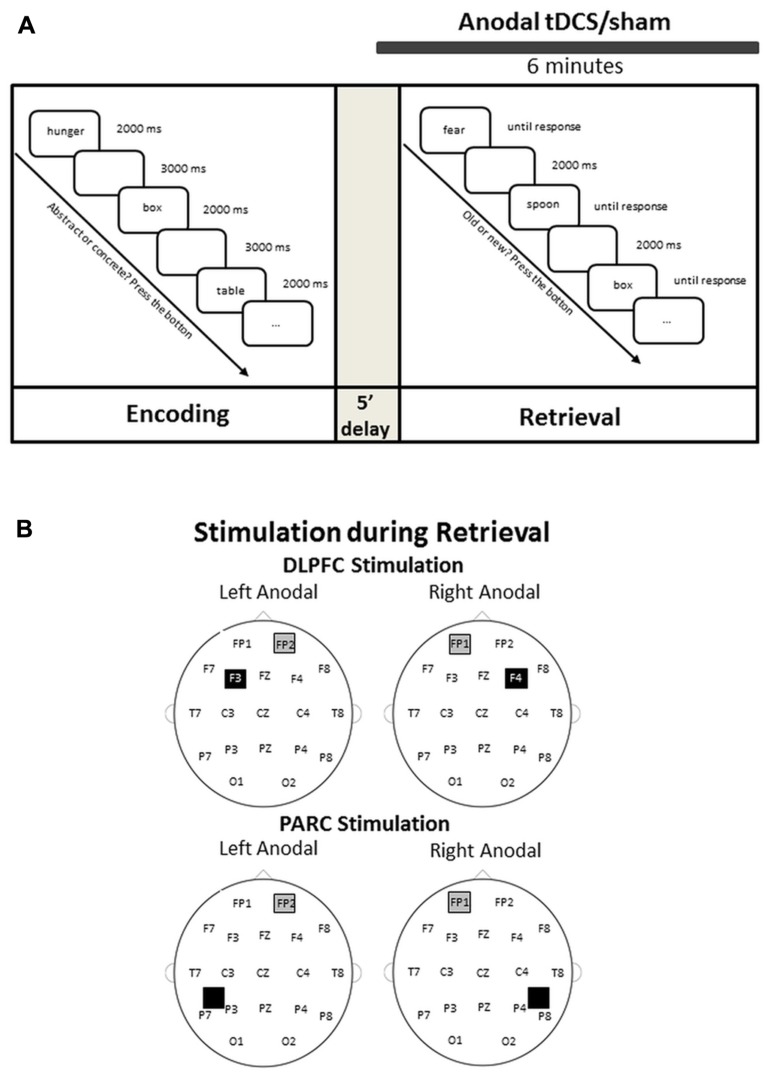
**(A)** Experimental design. An encoding phase was followed by a retrieval phase. tDCS was applied for 2 min before retrieval and throughout the retrieval. **(B)** Electrode montage on the dorsolateral prefrontal cortex (DLPFC) and on the parietal cortex (PARC).

The experiment design is illustrated in **Figure 1**.

#### tDCS procedure

The stimulation was delivered by a battery-driven, constant current stimulator (BrainStim, EMS, Bologna, Italy) through a pair of saline-soaked sponge electrodes (7 cm × 5 cm). A constant current of 1.5 mA was applied for 6 min (with a ramping period of 10 s at the beginning and at the end of the stimulation), starting 2 min before the beginning of the retrieval task and lasting for the entire retrieval phase. The current density (0.043 mA/cm^2^) was maintained below safety limits (Poreisz et al., 2007). The electrodes were secured using elastic bands, and to reduce contact impedance, an electroconductive gel was applied under the electrodes before the montage. In the two age groups, each participant was randomly assigned to either PARC or DLPFC stimulation, yielding two young groups (16 PARC stimulation and 16 DLPFC stimulation) and two older groups (16 PARC stimulation and 16 DLPFC stimulation). The study was a randomized single-blind experiment: the subjects did not know which stimulation they received, but the experimenter did. The three stimuli blocks corresponded to three stimulation conditions: anodal left, anodal right and sham (i.e., placebo).

In the DLPFC groups, the active electrode was placed on the left or right, 8 cm frontally and 6 cm laterally with respect to the scalp vertex; in the PARC groups, the active electrode was placed 5 cm posteriorly and 8 cm laterally with respect to the scalp vertex. The reference electrode was fixed on the contralateral supraorbital area. In the sham stimulation, the tDCS montage was the same, but the current was turned off 10 s after the start of the stimulation and was turned on for the last 10 s of the stimulation period (plus the duration of the fade-in and fade-out periods = 10 s),

Therefore, subjects felt the itching sensations below the electrodes at the beginning and at the end of the stimulation, making this condition indistinguishable from the experimental stimulation. Potential tDCS side effects were assessed with a questionnaire (Fertonani et al., 2010) at the end of each session. The active stimulations (i.e., anodal left and anodal right) were executed on two different days to minimize the likelihood of interference effects. The sham stimulation was always performed before the active stimulation. For a schematic representation of the full list of conditions used, see **Table 3**.

**Table 3:** Experimental conditions.

Stimulation site	First session	Second session
DLPFC	Sham-anodal right	Anodal left
	Sham-anodal left	Anodal right
	Anodal right	Sham-anodal left
	Anodal left	Sham-anodal right
PARC	Sham-anodal right	Anodal left
	Sham-anodal left	Anodal right
	Anodal right	Sham-anodal left
	Anodal left	Sham-anodal right

*Data analysis. Statistical analyses were performed using Statistica software (version 10; ) and SPSS (Version 21.0, IBM SPSS Statistics for Windows. Armonk, NY: IBM Corp).

For each age category (young and older), demographic variables (e.g., age and education) were compared between the two stimulation groups (DLPFC and PARC) using parametric (*t*-test) and non-parametric (Mann–Whitney test) analyses. Moreover, *t*-tests were conducted to compare both subjective sensations induced by the different tDCS protocols and the performance acquired during encoding in the three experimental blocks.

Behavioral data were analyzed for both accuracy and RTs during the retrieval sessions. Accuracy data were analyzed using signal detection theory. For each participant, the *d* prime (*d*′) for sensitivity to the previously seen words was estimated (Macmillan and Creelman, 2005). The correct recognition of a previously seen word constituted a hit, while erroneous recognition of a “new” word as an “old” word constituted a false alarm (FA). Hit and FA rates were transformed to *Z* scores using the standard normalized probability distribution. The *d*′ was estimated as the difference between the standardized scores (*Z*) of the hit rates (*H*) and of the FA rates.

A generalized estimating equations (GEE) model (Hardin and Hilbe, 2003) was adopted to analyze the non-normal (Gamma distributed) dependent variable RT measured according to the experimental design including two within factors: stimulation (left anodal, right anodal and sham), word categories (abstract and concrete); and two between factors: stimulated areas (DLPFC and PARC) and recruited groups (older and young). With the same experimental design, an ANOVA model was performed for the dependent variable accuracy.

Bonferroni corrections were adopted for all comparison adjustments of *post hoc* analyses.

## Results

No differences in age or education were observed between the stimulation groups in either the young or older subjects.

We also looked for performance differences between blocks during encoding. Block number had no effect on accuracy or RT for either abstract or concrete words, suggesting that the word lists in the three blocks required similar concreteness judgment during encoding.

### Stimulation Questionnaire

Perceptual sensations induced by the anodal tDCS and sham tDCS conditions were assessed with standardized questionnaire developed by Fertonani et al. (2010). Participants were asked to evaluate intensity of several perceptual sensations (i.e. itching, pain, burning, heat, pinching, iron taste, fatigue, effect on performance) through a 5-point-scale (0 = none, 1 = mild, 2 = moderate, 3 = considerable, and 4 = strong).

By interpreting the questionnaire completed by all subjects at the end of each type of stimulation we inferred that all the subjects well tolerate the stimulation and reported only marginal perceptual sensations. Itch and irritation were the most commonly reported perceptual sensations, with light to moderate intensity. Overall, the experienced perceptual sensations started at the beginning of the experiment, did not last long and did not affect task performance in the anodal or sham conditions. For each group (young and older participants) and each area (DLPFC or PARC), the sensations scores reported during the left and right anodal tDCS were compared with the sensations reported during the sham tDCS by a single-tailed paired *t*-test. These analyses showed that the anodal stimulations could not be distinguished from the sham stimulation [Young subjects: left DLPFC vs. sham DLPFC, *t* = -1.58, df = 15, *p* = 0.14; right DLPFC vs. sham DLPFC, *t* = -0.85, df = 15, *p* = 0.41; left PARC vs. sham PARC, *t* = 1.57, df = 15, *p* = 0.14; right PARC vs. sham PARC, *t* = 1.84, df = 15, *p* = 0.09; and Older subjects: left DLPFC vs. sham DLPFC, *t* = 1.54, df = 15, *p* = 0.15; right DLPFC vs. sham DLPFC, *t* = 0.24, df = 15, *p* = 0.82; left PARC vs. sham PARC, *t* = 1.29, df = 15, *p* = 0.22; right PARC vs. sham PARC, *t* = 0.25, df = 15, *p* = 0.81]. There are no reasons to reject the single-blinded character of this study on the basis of these results.

#### Reaction Time Analysis

***General tDCS effects***. The GEE model that included three types of stimulation (left anodal, right anodal, or sham-placebo, within subjects), two word categories (abstract or concrete, within subjects), two stimulated areas (DLPFC or PARC, between subjects), and two age groups (older or young, between subjects) as factors, revealed significant effects for age (Wald Chi-squared χ^2^ = 176.15, df = 1, *p* < 0.001), type of stimulation χ^2^ = 28.84, df = 2, *p* < 0.001), word category (χ^2^ = 122.72, df = 1, *p* < 0.001) and the interaction between stimulated areas and the word category (χ^2^ = 4.21, df = 1, *p* < 0.040). No significant effect was found for the interaction between word category and type of stimulation (χ^2^ = 0.44, df = 2, *p* < 0.801).

*Post hoc* analyses (pairwise comparisons of estimated marginal average carried out by Bonferroni adjustment) indicated that older subjects had slower RTs than young individuals (994 ms, 95% CI [933–1060] vs. 787 ms, 95% CI [751–825], *p* < 0.001); abstract words induced longer RTs than concrete words (922 ms, 95% CI [883–962] vs. 849 ms 95% CI [817–881], *p* < 0.001), and a general facilitation was induced by left tDCS application (left tDCS = 851 ms, 95% CI [819–884] vs. placebo tDCS = 911 ms, 95% CI [870–954], *p* < 0.001; and left tDCS vs. right tDCS = 892 ms, 95% CI [855–930], *p* = 0.002).

**FIGURE 2:**
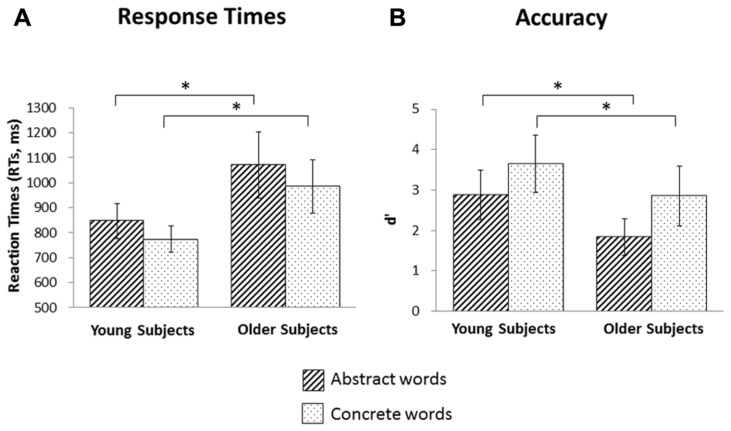
**Behavioral effects. (A)** Reaction times for abstract and concrete words in the young and older groups. Abstract words induced longer reaction times both in young (*p* = 0.001) and in older (*p* = 0.001) subjects. Moreover, older subjects were slower than young subjects in both abstract (*p* = 0.0001) and concrete words (*p* = 0.0001). **(B)** Accuracy for abstract and concrete words in the young and older groups. Abstract words induced worse accuracy in both young (*p* = 0.0001) and in older (*p* = 0.0001) subjects. Moreover, older subjects performed worse than young subjects in both abstract (*p* = 0.0001) and concrete words (*p* = 0.0001). Asterisks indicate significant effects (*p* < 0.05).

***Behavioral effect in young and older subjects.** Post hoc* pairwise comparisons, evaluated conditionally on sham stimulation, indicated that abstract words induced longer RTs than concrete words in both young (abstract = 847 ms vs. concrete = 773 ms, *p* < 0.001) and older subjects (abstract = 1070 ms vs. concrete = 985 ms, *p* < 0.001). Moreover, significant differences were found among the two type of words across age categories (for abstract words: young vs. old, *p* = 0.001; for concrete words: young vs. old, *p* < 0.001); see, e.g., **Figure 2A**.

**FIGURE 3:**
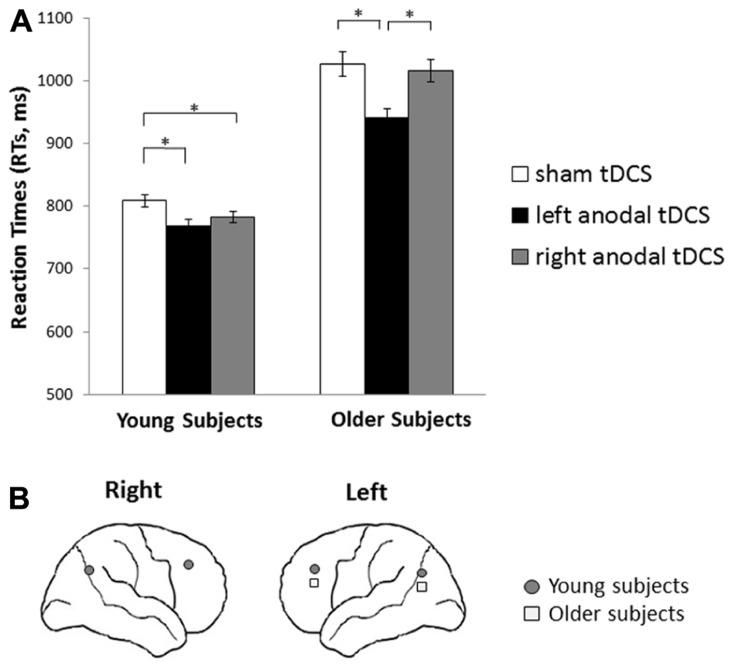
**Transcranial direct current stimulation effects on reaction times in young and older subjects during retrieval phase. (A)** Reaction times (RTs) achieved during retrieval of words by young and older subjects following tDCS applied over dorsolateral prefrontal cortices (DLPFCs) and parietal cortices (PARCs) compared to sham stimulation. Older participants were consistently faster during left stimulation than during sham stimulation. Young participants were consistently faster during left and right stimulation than during sham stimulation. Asterisks indicate significant effects (*p* < 0.05). **(B)** Graphical representation of the cerebral areas (DLPFCs and PARCs) related to a reduction of reaction times following anodal tDCS in young and older subjects.

***tDCS effect in young and older subjects***. A general facilitation was induced by left tDCS application in older subjects (left tDCS = 942 ms vs. placebo tDCS = 1027 ms, *p* < 0.001; and left tDCS vs. right tDCS = 1016 ms, *p* = 0.003); whereas in young participants, only placebo tDCS (809 ms) differed from right tDCS (783 ms, *p* = 0.050) and from left tDCS (769 ms, *p* = 0.026). See **Figures 3A, B** for details.

#### Accuracy Analysis

***General tDCS effects***. An ANOVA that included three types of stimulation (left anodal, right anodal, or sham, within subjects), two word categories (abstract or concrete, within subjects), two stimulated areas (DLPFC or PARC, between subjects) and two age groups (older or young, between subjects) as factors showed significant effects for age (*F*_1__, 6__0_ = 23.764; *p* = 0.000008), word category (*F*_1__, 6__0_ = 35.390; *p* = 0.000000), and the interaction between the type of stimulation and the word category (*F*_2,1__20_ = 4.089; *p* = 0.019). *Post hoc* analyses (Fisher’s least significant difference, LSD) showed that older subjects achieved lower accuracy than young individuals (2.28 ± 1.2 vs. 3.09 ± 1.3, *p* = 0.000008), abstract words induced worse performance than concrete words (2.31 ± 1.2 vs. 3.06 ± 1.3, *p* = 0.000000) and left tDCS application induced a general interference in concrete word recognition (left tDCS = 2.8 ± 1.1, placebo tDCS = 3.3 ± 1.5, *p* = 0.000003). No other effects were statistically significant. See **Figure 2B** for details.

## Discussion

Memory is the capacity to store, maintain, and retrieve events or information from the mind. Successful remembering implies a correct encoding and an appropriate retrieval of the information. Overall, our results demonstrated that anodal tDCS applied during the retrieval phase facilitates verbal episodic memory in young and in older subjects. In particular, we found that tDCS applied over the left and right regions (DLPFC and PARC) induces better performance in young participants; only tDCS applied over the left regions (DLPFC and PARC) increased retrieval in older subjects. Remarkably, these facilitation effects were observed during retrieval of both abstract and concrete words.

In agreement with the literature, we found that older subjects experience a significant decline in verbal episodic memory compared to young subjects. Furthermore, our findings suggest that non-invasive brain stimulation, in particular anodal tDCS, applied to left regions could be useful in enhancing memory function in aging. This result agrees with neuroimaging studies that demonstrated an age-related decrease in retrieval that occurred in several regions, including right prefrontal areas and right parietal regions (Grady et al., 1995; Schacter et al., 1996; Cabeza et al., 1997).

Physiological aging induced structural and functional changes have been linked to residual brain plasticity to counteract neural loss (Jancke, 2009). It has therefore been suggested that neural plasticity facilitates alternative “strategies” to maintain an adequate level of cognitive performance (Greenwood, 2007; Zollig and Eschen, 2009; Cotelli et al., 2010, 2012). The significance of these changes is intriguing because they could be caused by either an effective functional compensation strategy or an inadequate and/or less efficient processing strategy.

Our data appear to be in line with lesion and functional imaging studies that have indicated that episodic memory involves a widespread network of brain structures, including the PFC and PARCs (Nyberg et al., 2000; Cabeza et al., 2008). Several reviews suggest that both encoding and retrieval are associated with activation in the medial-temporal, prefrontal, and parietal regions. The left ventrolateral PFC and the medial-temporal regions are strongly involved in encoding, whereas the left superior parietal and the dorsolateral and anterior PFC regions are more strongly engaged in retrieval (Spaniol et al., 2009).

Consistent with the HERA model, we observed right involvement during retrieval in young individuals. Moreover, verbal memory retrieval performance was also facilitated when anodal tDCS was applied to left cortical regions (in either young or old individuals). This finding may be consistent with a material specific-model, which postulates that the left hemisphere is engaged in verbal memory processes and the right hemisphere is involved in visuo-spatial memory processes (Wagner et al., 1998; Golby et al., 2001). Furthermore, the asymmetrical left facilitation observed in old subjects was interpreted as reflecting a loss of regional specialization or declining specificity, referred to a dedifferentiation process, which has been hypothesized to occur in physiological aging (Park et al., 2004; Park and Reuter-Lorenz, 2009; Goh et al., 2010).

To investigate the effects of the two different types of encoded material, we compared abstract and concrete word retrieval performance during tDCS. We failed to observe any tDCS difference in abstract and concrete words. In particular, our results revealed that in young and older subjects, both abstract and concrete word retrievals were facilitated by DLPFC and PARC stimulation. The representation of abstract and concrete concepts is an open question in cognitive neuroscience (Kiefer and Pulvermuller, 2012). Neuroimaging studies do not provide converging evidence for neural correlates of abstract and concrete words, suggesting a bilateral representation for concrete words and a less defined network (left, right or bilateral) for abstract words (Kiehl et al., 1999; Fiebach and Friederici, 2004; Sabsevitz et al., 2005; Manenti et al., 2010; Rodriguez-Ferreiro et al., 2011). We interpreted the selective involvement of the left areas during the retrieval of abstract and concrete words in older individuals as an expression of a primary use of verbal code and an inefficient mental imagery strategy. This hypothesis is consistent with the idea that the capacity to generate non-verbal mental image strategies declines with age (Johnson and Rybash, 1993; Dror and Kosslyn, 1994; Manenti et al., 2011).

The tDCS technique involves the application of weak electrical currents (~0.5–2 mA) directly to the head for several minutes (~5–20 min; Priori, 2003; Nitsche et al., 2008). These currents generate an electrical field that modulates neuronal activity according to the modality of the application. Neurons respond to tDCS by altering their firing rates. Cathodal polarization over the motor cortex can induce reductions in motor cortex excitability, while anodal polarization increases motor cortex excitability (Nitsche and Paulus, 2000). These changes last for minutes to hours beyond the end of the stimulation, depending on the stimulation parameters. Both long-term potentiation and its opposite, long-term depression, have also been postulated to explain the persistent effects of non-invasive brain stimulation on cortical activity (Cooke and Bliss, 2006; Thickbroom, 2007; Ziemann and Siebner, 2008). The long-term effect is a crucial issue for the potential application of these techniques into rehabilitation intervention to ameliorate cognitive deficits.

In conclusion, anodal tDCS can be a relevant tool to modulate the long-term episodic memory capacities of young and older subjects. Memory declines with physiological aging, and memory loss is a characteristic of several clinical conditions. These preliminary findings suggest that anodal tDCS is able to modulate memory performance; this technique could be an interesting approach to study functional adaptation during physiological aging and eventually it could be employed to attempt to reduce the cognitive deficits observed in pathological brain aging.

## Conflict of Interest Statement

The authors declare that the research was conducted in the absence of any commercial or financial relationships that could be construed as a potential conflict of interest.
